# Editorial: Insights in zoological medicine: 2023

**DOI:** 10.3389/fvets.2025.1603605

**Published:** 2025-05-07

**Authors:** Irene Iglesias, Carlos Sacristan

**Affiliations:** Epidemiology and Environmental Health Group, Animal Health Research Center, National Institute for Agricultural and Food Research and Technology, Madrid, Spain

**Keywords:** wildlife conservation, environmental and anthropogenic factors, infectious diseases, captive animal welfare, disease surveillance

Over the past decade, remarkable progress has been made in zoological medicine through bold research initiatives and the application of innovative technologies. As we enter the third decade of the 21st century, it is crucial to understand not only the achievements but also the persistent and emerging challenges faced by wildlife in both natural ecosystems and human-altered environments. In the article series “*Insights in zoological medicine: 2023,”* we highlight the importance of wildlife studies in advancing knowledge on the Research Topic affecting these species and the interactions between wildlife, the environment, and human activities, emphasizing both achievements and outstanding challenges.

The studies by Tummeleht et al. and Haman et al. are notable examples of the interaction between wildlife and environmental risks, as well as the importance of early intervention and monitoring strategies. The first study examines the attraction of wild boar (*Sus scrofa*) carcasses to scavenging animals, which may serve as potential sources of African swine fever virus (ASFV) contamination in the environment. This research reveals the complexity of ASFV transmission routes, once interactions between wildlife —such as hooded crows (*Corvus cornix*) and red foxes (*Vulpes vulpes*)—and infected carcasses could facilitate the virus' spread. Additionally, the study underscores the need for proper carcass management to mitigate disease propagation among wildlife and in swine farming. Haman et al. investigated the significant mortality caused by the highly pathogenic avian influenza virus (HPAIV) H5N1 among seabirds (devastating bird colonies) and mammals along the Pacific coast of North America. Their findings highlight the necessity of coastal environment monitoring to better understand HPAIV epidemiology and mitigate its impact on vulnerable bird and mammal species.

The previous examples illustrate how human interventions can help wildlife conservation; however, human actions can also severely compromise the health and wellbeing of wild animals, particularly those kept in inadequate captive conditions. A pertinent example is the study by Stagni et al., which identified significant health problems in brown bears (*Ursus arctos*) rescued from suboptimal captivity. This retrospective research highlights common diseases and clinical abnormalities in these bears, including oral cavity and digestive system disorders, associated with the maintenance conditions before their rescue. Bears rescued from circuses and restaurants exhibited a higher prevalence of certain conditions, reinforcing the need for veterinary and captive management plans to improve their welfare. In another study conducted at a rescue center in Vietnam, Ludi et al. analyzed foot-and-mouth disease outbreaks affecting bears, demonstrating how diseases can rapidly spread in confined spaces and emphasizing the need for surveillance and preventive management in these environments.

Another critical issue regarding captive wildlife is antimicrobial resistance. The study by Kim et al. explores the prevalence of bacterial resistance in animals housed in zoos. The detection of multidrug-resistant *E. coli* and *Enterococcus faecalis* suggests potential bacterial exchange between animals and humans, a significant concern for public health and captive wildlife management. Additionally, continuous animal welfare assessment in zoos is a fundamental concern, as explained by Liptovszky. Advanced data science techniques, such as syndromic surveillance (1) and comprehensive diagnostic examinations, as demonstrated by Vetere et al., can significantly enhance the quality of life of captive animals by ensuring efficient resource allocation and improvement of ongoing care and management.

Another study focusing on infectious agents in wildlife is the one by Carella et al., on the potential immunosuppressive effect of picornaviruses in the noble pen shell (*Pinna nobilis*)—a marine mollusk, illustrating the severe threat that viral pathogens pose to endangered species. This virus may cause mass mortality events in the Mediterranean Sea, highlighting the urgency of incorporating virological studies into marine conservation efforts to identify and mitigate similar risks.

Human activities also impact wildlife through climate change effects, as demonstrated in the study by Guan et al. on the gastrointestinal microbiome of three-toed box turtles (*Terrapene carolina triunguis*). This study suggests that the microbiome of this species may be resilient to rapid temperature changes, although the increased presence of pathogens such as *Erysipelothrix* sp. at higher temperatures could have adverse effects.

In summary, these studies demonstrate the value of a multidisciplinary approach to advancing zoological medicine and understanding the challenges faced by wildlife. Moreover, these investigations raise critical questions regarding the effects of human activities and environmental changes on wildlife health. The Research Topic and analysis of environmental data, alongside wildlife health monitoring, are essential to elaborate effective responses to these challenges. Such efforts also build a foundation for the development and implementation of more effective wildlife conservation and management policies. By addressing these Research Topic, we are better prepared to anticipate and mitigate risks posed by human activities and habitat alterations over wildlife health, ultimately ensuring long-term biodiversity preservation and ecosystem health.
